# Hybrid Biochar from Corn Stover and Sewage Sludge for VOCs Adsorption: A Sustainable Waste Utilization Approach

**DOI:** 10.3390/toxics14060516

**Published:** 2026-06-12

**Authors:** Zhen Zhang, Ninglu Zhang, Xiaohui Pan, Bingchao Zhao, Jun Liu, Shujian Tian, Liyu Hao, Zihao Zhao

**Affiliations:** 1 College of Energy and Power Engineering, North China University of Water Resources and Electric Power, Zhengzhou 450046, China; 2 College of Mechanical & Electrical Engineering, Henan Agricultural University, Zhengzhou 450002, China

**Keywords:** sludge, corn stover, sludge-based biochar, adsorption, VOCs

## Abstract

Volatile organic compounds (VOCs) are major contributors to air pollution and pose significant risks to both environmental quality and human health. Biochar-based adsorption technology is an efficient and sustainable approach to VOCs removal. Herein, hybrid biochar was prepared from corn stover and municipal sewage sludge using the water vapor activation method, and its physicochemical characteristics and adsorption mechanisms for typical volatile organic compounds commonly produced during biomass-derived energy generation—such as methylbenzene, isopentane, and ethylene—were systematically investigated. The results show that hybrid biochar significantly outperformed single-source biochar, with its ability to adsorb methylbenzene, isopentane, and ethylene exceeding that of pure sludge biochar by 112.21%, 74.53%, and 66.72%, respectively, and surpassing pure corn stover biochar by 74.25%, 62.98%, and 55.25%, respectively. Competitive adsorption analysis indicated that the interaction strength between VOC molecules and the steam-treated hybrid carbon material was associated with their boiling points; compounds with higher boiling points tended to exhibit stronger affinity. This work provides an integrated waste utilization and pollution control strategy for VOCs removal.

## 1. Introduction

Volatile organic compounds (VOCs) are a major source of air pollution, posing severe risks to both the environment and human health [1]. According to a survey, the total volatile organic compounds (TVOC) concentration in Tianjin suburbs in summer is as high as 174 ppbv, which exceeds the general indoor air quality reference limit (about 160 ppbv) [2]. With ongoing industrialization and increased energy production, the release of volatile organic compounds has emerged as an escalating concern for air quality, particularly during the operation of energy facilities such as biomass power plants. These pollutants encompass hydrocarbons, halocarbons, and oxygenated compounds [3]. The removal of VOCs typically relies on two primary approaches: optimizing processes and equipment or implementing supplementary control technologies. Process and equipment modifications are often costly, thus limiting their applicability. In contrast, adsorption technologies, a key aspect of the control-based approach, provide high VOC removal efficiency and are gaining recognition as a promising solution [4].

Biochar is a sustainable, cost-effective adsorbent for VOC removal. Interest in utilizing biochar as an adsorbent for mitigating harmful gaseous pollutants is increasing. Traditional precursors for activated biochar, such as coal and pitch, are non-renewable and associated with high production costs, which limits their widespread application [5]. Ning Cheng et al. reviewed over 100 published studies, highlighting a significant difference in adsorption rates between biochar and activated biochar [6]. Biochar modification is gaining attention, with increased efforts to improve its physicochemical properties and adsorption capacity.

Biomass energy derived from organic materials plays a vital role in optimizing energy utilization, mitigating CO_2_ emissions, and supporting ecosystem conservation [7]. Driven by the pressing need for environmental protection and climate mitigation, China, as the world’s largest carbon emitter, has attached great importance to the development and utilization of biomass resources, especially energy crops [8]. Although numerous biomass sources are accessible for human utilization, current discussions and research have predominantly focused on agricultural and forestry residues as well as energy crops, while relatively less attention has been given to resources such as livestock waste, urban waste materials, and wastewater [9]. The conversion of sewage sludge and crop straw into biochar for VOC adsorption is highly practical and promising. Biochar derived from biomass waste not only offers effective removal of air pollutants, but also plays a role in promoting the circular economy through reducing greenhouse gas emissions and transforming waste into high-value carbon materials [10].

Numerous studies have investigated the adsorption characteristics of biochar. He, Shi, Gao et al. demonstrated that activation-induced increases in specific surface area and pore volume significantly enhance the adsorption capacity of activated biochar for gaseous benzene. These studies explored different preparation and activation conditions, highlighting that micropores with specific size distributions facilitate benzene adsorption [11,12,13]. Zhang, Aleksandra, et al. found that tuning the surface functionalities and adjusting the structure of activated biochar can effectively enhance its capacity to adsorb aromatic hydrocarbons, such as methylbenzene and benzene. These studies comprehensively examined how carbon materials with varying pore architectures interact with adsorbates, emphasizing how molecular polarity and structural traits influence both adsorption strength and capacity [14,15]. Separate studies by Azmatullah, Zhang, and Shih independently explored how activated biochar performs in removing benzene and other VOCs. Their findings indicated that physical adsorption predominates, and that the pore structure plays a vital role in improving adsorption kinetics [16,17,18]. Welham, Cheng, and Lashaki et al. analyzed the adsorption mechanism of activated biochar and found that milling enhances its chemisorption capacity. These studies identified active surface area, electron exchange between the adsorption medium and adsorbent forming chemical bonds, and monolayer adsorption as key factors influencing chemisorption [19,20,21]. Kottis et al. showed that blended biochars derived from multiple biomass sources typically have better surface area, porosity and electrical properties compared to single feedstock biochars [22]. Zhou et al. showed that activated biochar prepared by the steam activation method has a high specific surface area and excellent adsorption properties for volatile organic compounds [23].

Currently, research on the adsorption and removal of volatile organic compounds (VOCs) using biochar has primarily focused on the carbonization and application of single natural biomass or single sludge feedstocks; systematic exploration of the synergistic preparation of composite biochar from multi-source waste materials remains insufficient. At the same time, although methods such as chemical activation or metal doping can effectively enhance the performance of biochar, the complexity of these processes, potential secondary pollution, and high costs severely limit their feasibility for large-scale application. To address these limitations and achieve the dual goals of “treating waste with waste” and environmental sustainability, this study innovatively proposed and systematically implemented an integrated preparation strategy for “steam-activated sludge–straw mixed biochar.” This strategy not only efficiently couples two abundant waste resources—sludge (municipal solid waste) generated during wastewater treatment and agricultural straw (agricultural solid waste)—but also employs a simple-to-operate, environmentally friendly steam physical activation technique to produce composite biochar with excellent pore structure and surface chemical properties (see this study for detailed preparation methods). The VOCs in flue gas emitted from biomass power plants consist primarily of alkenes, alkanes, and aromatic hydrocarbons. Among these, ethylene accounts for the largest proportion of alkenes, isopentane is the predominant alkane, and methylbenzene is the predominant aromatic hydrocarbon, with each constituting more than 85% by mass [24]. Therefore, this study primarily focuses on ethylene, isopentane, and methylbenzene as the VOCs of interest. The VOC adsorption performance evaluation in this study targeted typical gases (methylbenzene, isopentane, ethylene) generated during biomass energy production. Through systematic research, we not only confirmed that the composite biochar prepared by this method exhibits significantly enhanced adsorption performance for single-component VOCs compared to biochar derived from a single raw material (e.g., pure sludge biochar or pure corn stover biochar) (see experimental results), but also systematically revealed the intrinsic mechanisms underlying the competitive adsorption of VOC mixtures by this material. These research findings will provide important theoretical and practical foundations for the development of low-cost, highly efficient, and environmentally friendly VOC adsorbents, offering new insights for promoting the synergistic development of waste resource utilization and air pollution control technologies.

## 2. Materials and Methods

### 2.1. Materials Used in the Experiments

The sewage sludge used in this study originated from the Zhengzhou Matougang Wastewater Treatment Plant, China.

**Figure 1:**
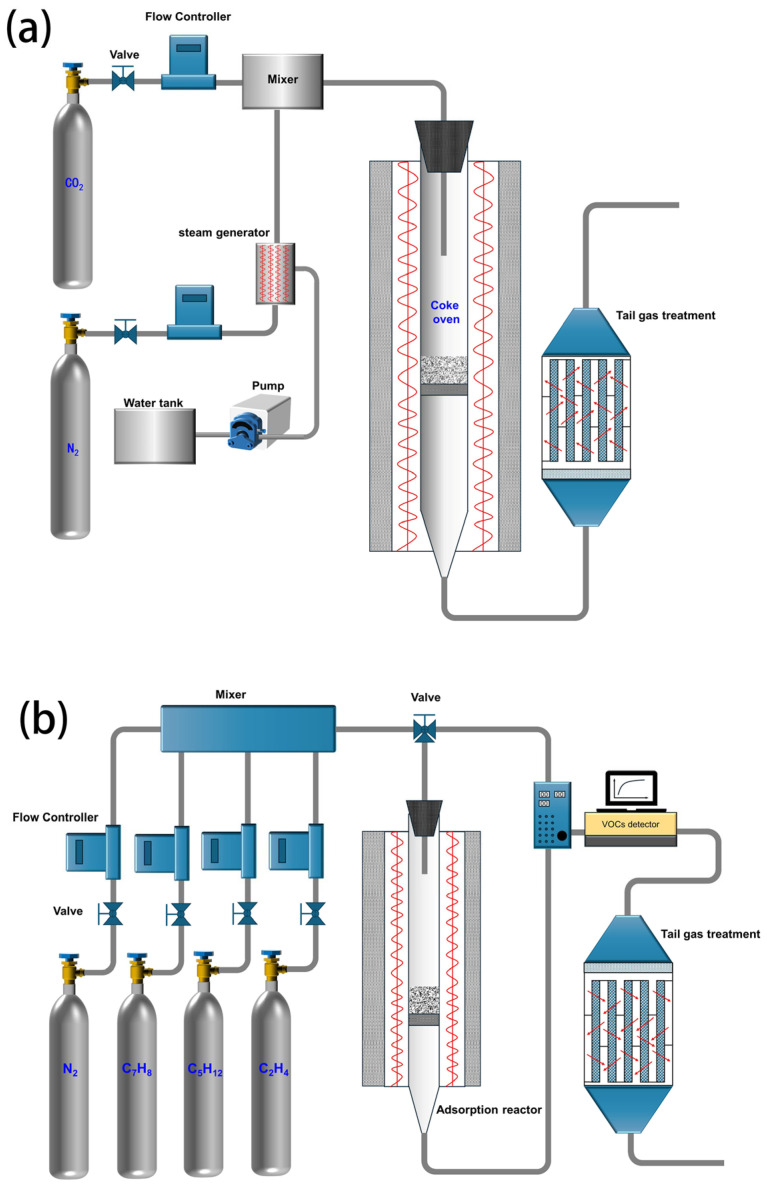
(**a**) Schematic diagram of the biochar production equipment. (**b**) Schematic diagram of the VOC adsorption test device.

Corn stover was sourced from the experimental farmland of Henan Agricultural University, China. The gas cylinders of the experimental setup were standard gas mixtures (8L, Jining Xieli Special Gas Co., China), and the volume fraction of methylbenzene, isopentane and ethylene in each cylinder was 100 ppm. The experimental setup consists of three parts: a gas unit, an adsorption unit, and a monitoring unit. The system is equipped with a pressure-reducing valve, a mass flow meter, and piping made of PTFE. The experimental setup is illustrated in Figure 1.

### 2.2. Experimental Methods

The sludge and corn stover mixture was heated to 600 °C at 10 °C/min under a nitrogen atmosphere (Cylinder pressure 0.1 MPa, flow rate 300 mL/min), held for 120 min, and cooled naturally to produce virgin biochar. Water vapor was introduced during the activation stage to obtain activated biochar under set conditions (Table 1). At the start of the experiment, 1.5 g of biochar was weighed. The biochar was then heated in a stream of N_2_ at 200 °C for 2 h to remove adsorbed impurities (primarily moisture), ensuring that the adsorption experiment was conducted in a dry environment. The adsorption experiment began after the sample had cooled to room temperature. As the adsorption temperature increased, the VOC adsorption capacity gradually decreased. In this study, the adsorption experiments were conducted in a constant-temperature apparatus maintained at 25 °C. The adsorption experiment employed an online VOCs detector that continuously tracked gas concentration levels throughout the process. Prior to the start of the experiment, the system was purged with nitrogen to remove air from the reactor until the oxygen concentration and relative humidity at the outlet dropped to zero. A mixture of VOCs was then introduced, and the experiment began once the gas concentration at the outlet had stabilized. The inlet gas flow rate was 500 mL/min, and the experiment was ended after adsorption equilibrium. For the binary adsorption experiments, methylbenzene–isopentane, methylbenzene–ethylene, and isopentane–ethylene mixtures with varying concentration levels (10, 30, 60 ppm) were used with the same monitoring method [25]. The data were recorded continuously during the experiment. Unactivated biochar has already been analyzed in the authors’ previous research; for details, see Reference [25]. The analytical results for unactivated biochar are directly cited in this paper.

### 2.3. Test Methods for Sample Properties

#### 2.3.1. Testing and Analyzing Methods for Specific Surface Area and Diameter of Hole

The specific surface area and pore structure of the biochar were determined by nitrogen adsorption using the Brunauer–Emmett–Teller (BET) and Barrett–Joyner–Halenda (BJH) methods. Before testing, samples underwent a 5-h degassing treatment at 200 °C in a strong vacuum environment (<0.01 mbar). Measurements were conducted at relative pressures ranging from 0.1 to 1 under liquid nitrogen at 77 K.

#### 2.3.2. Test Methods for Functional Groups

Fourier transform infrared spectroscopy (FTIR) is an efficient and economical technique for the qualitative and quantitative analysis of organic and inorganic components in gases, liquids, and solids. In this experiment, biochar was mixed with KBr at a ratio of 1:100 and pressed into pellets. The samples were then subjected to 32 scans in the range of 400–4000 cm^−1^ using an spectrometer (IR-960, Tianjin Tianguang Optical Instrument Co. Ltd., Tianjin, China) to obtain high-resolution spectral data.

#### 2.3.3. Scanning Electron Microscopy (SEM)

The synthesized biochar’s external microstructure was examined and analyzed at room temperature using scanning electron microscopy (SEM) (Zeiss Sigma 300, Oberkochen, Germany). This technique enabled detailed microstructural analysis, revealing the morphological characteristics of the samples.

### 2.4. Response Surface Method Experimental Design

The optimal water vapor activation method for SBC was identified using the Box–Behnken model in Design Expert software (version 14). [26]. Regression equations were developed and validated using test point data, leading to the identification of optimal process parameters. Experimental factors and their corresponding levels are presented in Table 2 and Table 3, with label explanations in Table 4.

### 2.5. Dynamics Analysis

Adsorption kinetic studies serve two purposes: (1) estimating the adsorption kinetics of volatile organic compounds using biochar derived from sewage sludge and (2) elucidating the adsorption mechanism [27]. The adsorption kinetic curves were fitted using pseudo-first-order and pseudo-second-order models.

Quasi-primary kinetic model:

$$\ln(q_{e}-q_{t})=\ln q_{e}-k_{1}t$$

Quasi-secondary kinetic modeling:

$$\frac{t}{q_{t}}=\frac{1}{k_{2}q_{e}^{2}}+\frac{t}{q_{e}}$$

where *k*_1_ (min^−1^) and *k*_2_ [(g·mg)^−1^·min^−1^] are the adsorption rate constants; and *q_e_* (mg·g^−1^) and *q_t_* (mg·g^−1^) denote the amount of adsorption at the adsorption equilibrium and at the moment of reaction t, respectively.

### 2.6. Adsorption Isotherms

The adsorption isotherm is the most effective model for evaluating adsorbent performance. The Langmuir equation is applicable to monolayer adsorption on surfaces with homogeneous adsorption sites [28]. The Freundlich model is particularly suited for multilayer adsorption on heterogeneous surfaces. The adsorption of methylbenzene, isopentane, and ethylene on biochar can typically be described by both the Langmuir and Freundlich models.

Langmuir model:

$$\frac{\rho_{e}}{q_{e}}=\frac{\rho_{e}}{q_{m}}+\frac{1}{k_{L}q_{m}}$$

Freundlich model:

$$\ln q_{e}=\ln k_{F}+\frac{\ln\rho_{e}}{n}$$

In the formula, *q_e_* and *ρ*_*e*_ represent the amount adsorbed at adsorption equilibrium (mg/g) and the concentration of inhaled gas (mg/m^3^), respectively; *q*_m_ indicates the maximum theoretical adsorption capacity (mg/g); *k_L_* denotes Langmuir’s adsorption rate constant (L/mg); *k_F_* expressed as Freundlich adsorption constant (mg·g^−1^·(L·mg^−1^)^−1/n^); and n is the strength of the adsorption.

## 3. Results and Discussion

### 3.1. Features of Biochar

#### 3.1.1. Parameter Optimization Using Response Surface Methodology

Based on the experimental results of methylbenzene, isopentane and ethylene adsorption (Table 2 and Table 3), quadratic regression model Equations (1)–(3) were obtained to express the effects of the variables on the amount of methylbenzene adsorbed, isopentane adsorbed and ethylene adsorbed:

(1) $$q_{e1}=254.60+24.54A+12.10B+6.68C+10.47AB+1.60AC-3.14BC-36.46A^{2}-22.36B^{2}-9.43C^{2}$$

(2) $$q_{e2}=220.96+14.10A+8.08B+5.27C+0.95AB+1.85AC-3.70BC-28.05A^{2}-9.65B^{2}-4.48C^{2}$$

(3) $$q_{e3}=195.41+7.16A+6.65B-0.034C+1.37AB+5.22AC+2.88BC-16.04A^{2}-2.85B^{2}-7.32C^{2}$$

where *q*_*e*1_~*q*_*e*3_ are the estimated responses of methylbenzene adsorption, isopentane adsorption and ethylene adsorption, respectively.

Analysis of variance (ANOVA) of the established models indicated that the regression models for the adsorption of toluene, isopentane, and ethylene were all highly significant (*p* < 0.001). A comparison of the *p*-values, F-values, and sums of squares revealed that the feedstock ratio and activation temperature had a significant effect on the VOC adsorption capacity of SBC, while the activation time had little effect. Furthermore, in toluene adsorption, the interactions between material ratio and activation time, material ratio and activation temperature, and activation temperature and activation time were all significant (*p* < 0.05). Other detailed ANOVA results are shown in Table 5.

As shown in Table 6, the correlation coefficients of the three models were large R^2^ > 0.9933, Adj R^2^ > 0.9846, Pred R^2^ > 0.9184, Adj R^2^ − Pred R^2^ < 0.2, C.V%. < 0.95%, suggesting that all three models are applicable for characterizing VOCs adsorption behavior (methylbenzene, isopentane and ethylene) by water vapor-activated SBC.

The ideal process parameters for VOCs adsorption using activated biochar were obtained according to the regression equations and are shown in Table 7. The results of the experiments were close to the predicted values, and the relative errors were all in the range of 0.84% to 2.26%, which makes this model reliable.

#### 3.1.2. Analysis of Surface Area and Pore Characteristics

**Figure 2:**
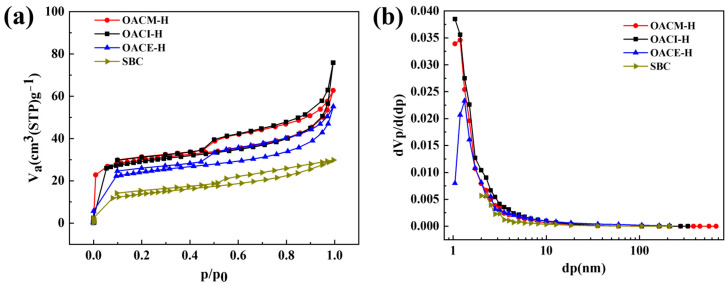
N_2_ Adsorption–desorption profile and pore architecture. (**a**) N_2_ adsorption–desorption curve, (**b**) pore size distribution plot.

Figure 2 presents the N_2_ adsorption–desorption isotherms and the pore distribution of the biochar. The results of Figure 2a showed that the unactivated SBC showed a type I adsorption isotherm, and the water vapor-activated biochar showed type IV. The rapid increase in adsorption at low pressure and the appearance of hysteresis loops in the desorption isotherms of the OAC group at high pressure indicate that the biochar has a mixed microporous and mesoporous structure and good adsorption properties, and the activation treatment removes the impurities and structure from the carbon source, making the pore structure of the carbon more complex.

The results of the analysis by the BJH method are shown in Figure 2b; the pore size of biochar is mainly distributed below 5 nm. The pore size range of both OAC and BC groups is 0–6 nm, of which about 80% of the pore size is microporous and 20% is mesoporous. The pore structure diagrams revealed the coexistence of micropores and mesopores in both biochar groups, which helped the biochar to adsorb molecules of varying dimensions.

Water vapor activation significantly improved the pore structure and specific surface area of SBC. As shown in Table 8, compared with pure sludge (82.53 m^2^/g) and pure CBC (80.71 m^2^/g), the specific surface area of the activated sample (OAC-H) amounted to 158.97 m^2^/g, which was an improvement of 92.62% and 96.70%, respectively, and 3.51 times higher than that of the unactivated sample (45.29 m^2^/g). The activation treatment also reduced the average pore size and increased the pore volume and porosity. This suggests that water vapor activation helps to remove organic components, expand the pore structure, and increase the effective adsorption sites, as well as promote the diffusion and transport of ions [29].

#### 3.1.3. Analysis of Surface Chemical Functionalities

**Figure 3:**
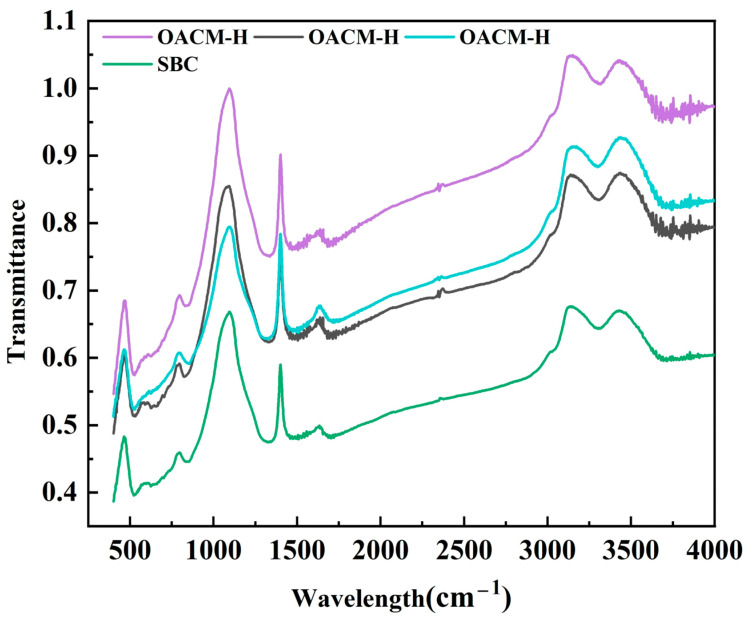
FTIR spectroscopy on biochar.

Figure 3 presents the functional group analysis results for SBC. Both water vapor-activated and unactivated SBC exhibited characteristic absorption peaks around 470, 790, 1110, 1400, 1630, 3200, and 3448 cm^−1^, reflecting similar functional group compositions. According to reference [30], these peaks and their corresponding functional groups are as follows: PO_4_^3−^ (P–O, 470 cm^−1^), NO_2_^−^ (N–O, 790 cm^−1^), C–O–C (1100 cm^−1^), CO_3_^2−^ (C–O, 1400 cm^−1^), C=C (1630 cm^−1^), and hydroxyl groups (–OH, 3200 and 3448 cm^−1^). The differences in absorption intensity indicate changes in surface functional group content during the activation process, particularly the enhancement of oxygen-containing groups (e.g., hydroxyl and carboxyl groups), which are closely related to VOCs adsorption. These groups enhance adsorption efficiency by enabling hydrogen bonds, charge-related interactions, and π–electron stacking effects, thereby strengthening the binding between biochar and VOC molecules [30,31]. Furthermore, the distribution and abundance of these groups are contingent on the pyrolysis temperature and activation conditions. These parameters directly impact the adsorption capacity of biochar [32].

#### 3.1.4. SEM Analysis

**Figure 4:**
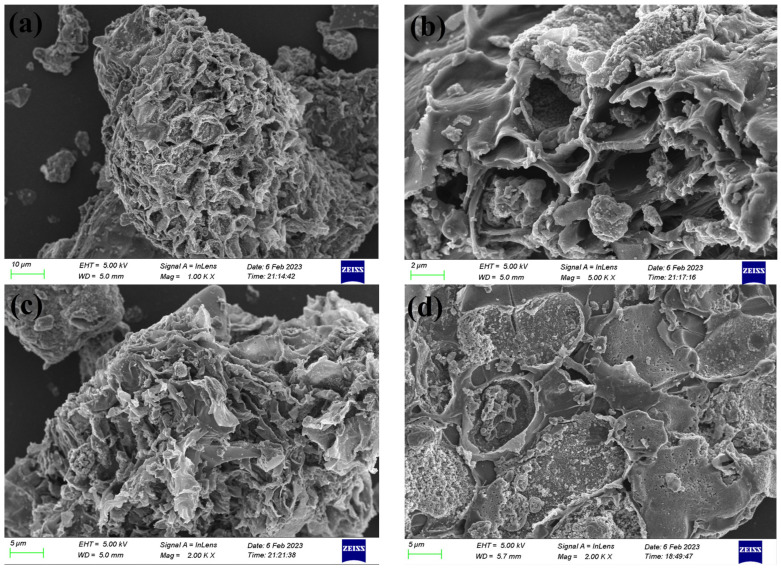
SEM image of the raw material. (**a**) OACM-H, (**b**) OACI-H, (**c**) OACE-H, (**d**) SBC.

The surface morphology of water vapor-activated biochar, along with its changes pre- and post-activation, was characterized through SEM imaging. The surface morphology of unactivated biochar is derived from our previous studies [25], as shown in Figure 4d, the unactivated biochar exhibited a comparatively smooth surface, featuring uneven and shallow pores with irregular geometry; in contrast, the surface of the water vapor-activated samples was significantly rougher with a denser pore distribution. Figure 4a–c demonstrates that the activation treatment promoted the development of the pore inner wall, forming more micropores with expanded pore diameters and increased fracture-like structures. By removing impurities from the pores and eroding the pore walls, the activation process effectively increased the ratio of micropores to mesopores, thus optimizing the pore structure and enhancing the adsorption performance of the biochar.

### 3.2. Adsorption Characterization of Sludge-Derived Biochar Produced via Water Vapor-Induced Activation

#### 3.2.1. Penetration Curves

**Figure 5:**
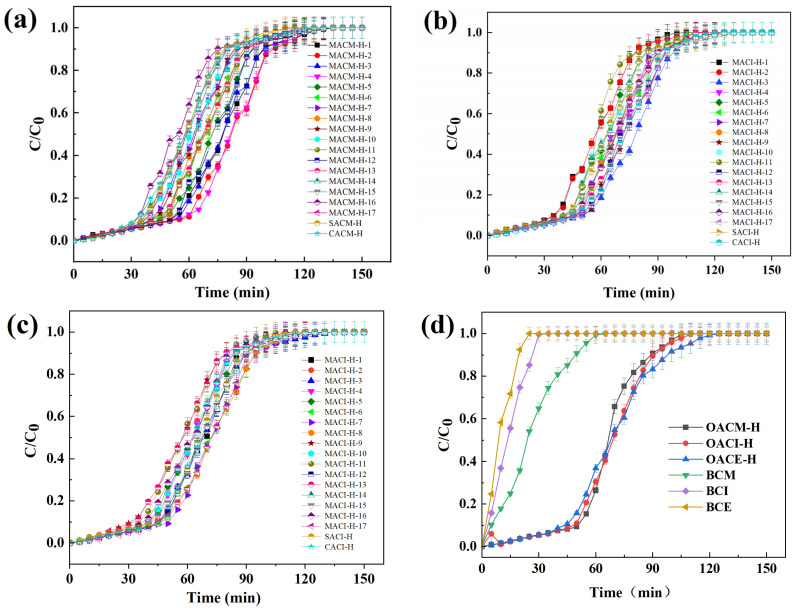
Penetration curve of SBC prepared by water vapor activation method for VOCs. (**a**) Methylbenzene, (**b**) isopentane, (**c**) ethylene, (**d**) best and unactivated groups.

SBC can effectively adsorb VOCs such as methylbenzene, isopentane, and ethylene. Breakthrough testing provides a direct method for evaluating its adsorption performance. Figure 5a–c shows the breakthrough curves for VOC adsorption by various groups, including water vapor-activated SBC and two groups of single-source biochar. Figure 5d compares the adsorption properties of OAC and BC.

The data in Figure 5a–c show that the breakthrough times for methylbenzene, isopentane, and ethylene were 35, 35, and 40 min, respectively, for pure sludge biochar (SAC-H). The corresponding times for pure CBC (CAC-H) were 30, 40, and 40 min. In comparison, most of the 17 response surface-designed SBC groups exhibited longer breakthrough times than the individual pure biochar groups. The optimal SBC identified through response surface analysis (Figure 5d) demonstrated superior adsorption capacity for various VOC species, with breakthrough times of 55, 50, and 45 min corresponding to methylbenzene, isopentane, and ethylene, and respective full-capacity adsorption durations of 105, 110, and 120 min. In contrast, the unactivated SBC (BC) showed breakthrough times of only 5 min for methylbenzene, isopentane, and ethylene, with corresponding saturation times of 55, 35, and 30 min, respectively. Among the evaluated materials, the sequence of optimal VOC uptake efficiency was as follows: OACM-H > OACI-H > OACE-H.

Based on the experimental results on pore structure in Section 3.1.1, steam activation led to a significant increase in the specific surface area, micropore volume, and mesopore volume of the sludge-based biochar. Van der Waals forces serve as the primary adsorption forces, and the well-developed hierarchical pore structure provides physical adsorption space for pore filling, thereby greatly enhancing physical adsorption. According to the results in Section 3.1.3, steam activation significantly enhanced the broad absorption peak at ~3200–3448 cm^−1^ on the biochar surface, which corresponds to hydroxyl (-OH) functional groups. According to the literature [30,31], hydroxyl functional groups provide biochar with more polar adsorption sites and also provide the structural basis for the formation of O-H···π or O-H···O type hydrogen bonds with polar/π-bonded VOC molecules, forming O-H···π or O-H···O type hydrogen bonds. Increasing the hydroxyl density on the adsorbent surface can significantly enhance hydrogen bonding. Therefore, the adsorption of VOCs by biochar is the result of the combined action of multiple physical adsorption mechanisms and chemical interactions.

#### 3.2.2. Adsorption Curves

**Figure 6:**
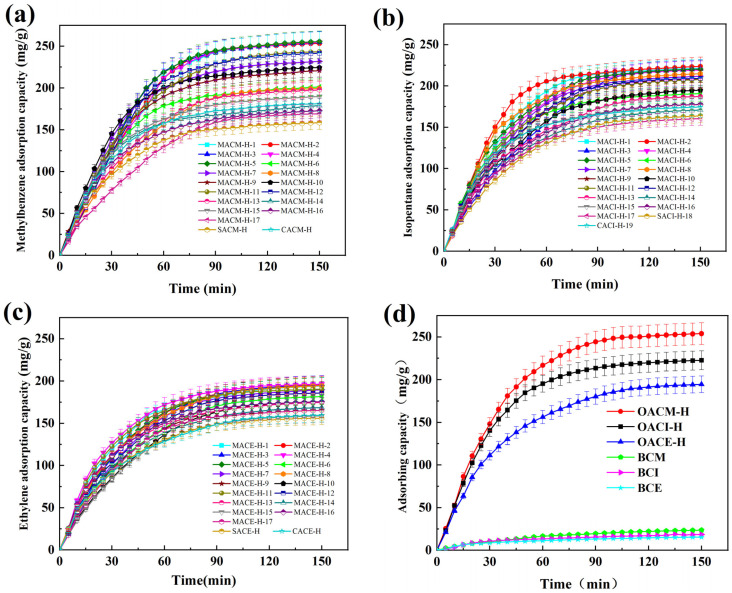
Adsorption curve of VOCs on SBC prepared by water vapor activation method. (**a**) Methylbenzene, (**b**) isopentane, (**c**) ethylene, (**d**) best and unactivated groups.

Figure 6 shows the contaminant adsorption profile of SBC prepared by steam activation. Figure 6a–c shows the adsorption capacity of volatile organic compounds with time for the biochar samples. Figure 6d compares the adsorption of volatile organic compounds by OAC and BC over time.

The adsorption of VOCs by water vapor-activated SBC followed a rapid-then-slow pattern, reaching equilibrium at approximately 150 min. The initial rapid adsorption was facilitated by the pore structure and electrochemical properties of the biochar. In addition, the presence of inorganic minerals and corn stover components synergistically enhanced the adsorption capacity. The synergistic effect of the mixed feedstocks significantly improved the removal efficiency of organic pollutants. As illustrated in Figure 6a, pure sludge biochar (SACM) exhibited the lowest capacity for methylbenzene adsorption. The adsorption of methylbenzene by pure CBC was only higher than the response surface 16 and 17 groups (MACM-16 and MACM-17), but lower than that of all other groups. As shown in Figure 6b, the isopentane adsorption capacity of SACI was higher than that of group 17, but lower than that of groups 1 to 16 and CACI, and the adsorption capacity of CACI was higher than that of groups 14, 17 and SACI. Figure 6c shows that the ethylene adsorption capacity of SACE was the lowest, followed by CACE, while the mixed biochar groups (MACE-1 to MACE-17) generally exhibited higher adsorption capacities than the single-feedstock groups. These results indicate that mixed-source biochar demonstrates superior performance in VOC adsorption. Figure 6d further illustrates that the adsorption capacities of VOCs by water vapor-activated SBC were significantly enhanced. Specifically, the adsorption amounts for OACM-H, OACI-H, and OACE-H reached 253.84 mg/g, 222.57 mg/g, and 194.63 mg/g, respectively. In contrast, the adsorption capacities of methylbenzene, iso-pentane, and ethylene by the unactivated biochar were only 23.62 mg/g, 18.57 mg/g, and 15.48 mg/g, respectively. After water vapor activation, the adsorption of these three VOCs increased by 10.74, 11.99, and 12.57 times, respectively. These findings indicate that water vapor activation substantially improved the pore structure and adsorption performance of SBC. The presence of water vapor facilitated pore formation, thereby enhancing the material’s capacity for VOC capture and storage [33].

#### 3.2.3. Saturated Adsorption Capacity

**Figure 7:**
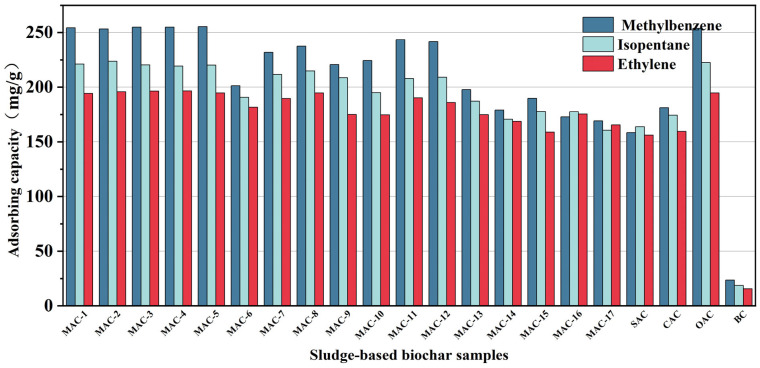
Adsorption of VOCs by water vapor-activated biochar samples.

The saturated adsorption capacities reported in this section (and presented in Figure 7) were determined from breakthrough tests (Section 3.2.1) under continuous-flow conditions, representing the dynamic total adsorption capacity of the fixed-bed column until saturation. This differs from the equilibrium adsorption capacities (qm) derived from the isotherm models (Section 3.2.5 and Table 8), which represent the theoretical maximum capacity under static batch adsorption at equilibrium. Figure 7 demonstrates the saturated adsorption of methylbenzene, isopentane and ethylene by unactivated and water vapor-activated SBC. It can be clearly seen that water vapor activation significantly increased the adsorption capacity of the biochar for the three VOC molecules. Furthermore, compared to the VOC adsorption capacities of bamboo, wood, sugar beet pulp, and sugarcane bagasse biochar reported in the literature [34], which range from 5.58 to 91.2 mg g^−1^, the adsorption capacity of the activated biochar in this study increased significantly. The adsorption of methylbenzene was increased by about 6.71 to 10.82 times, isopentane by 8.65 to 12.05 times, and ethylene by 10.09 to 12.71 times as compared to the BC group. The highest adsorption amounts were 255.46 mg/g (MAC-5 group), 223.74 mg/g (MAC-2 group) and 196.45 mg/g (MAC-4 group), respectively. In all cases, the MAC group exhibited markedly superior adsorption performance compared to the SAC group, with the maximum adsorption enhancement of 61.23%, 36.5% and 25.78% for methylbenzene, isopentane and ethylene, respectively. Meanwhile, most of the samples in the MAC group were also superior to those in the CAC group, with maximum enhancements of 40.99%, 28.25% and 23.07%, respectively. This improvement was primarily ascribed to the abundant carboxylic acid groups introduced onto the biochar surface through water vapor activation, which have strong adsorption capacity and can form complexes with VOCs, so that methylbenzene, isopentane, and ethylene were effectively bound on the surface of the biochar. Furthermore, water vapor activation increased the specific surface area and enhanced the interaction between adsorbent and adsorbate, which significantly improved the adsorption efficiency of VOCs [35].

#### 3.2.4. Kinetic Analysis

**Figure 8:**
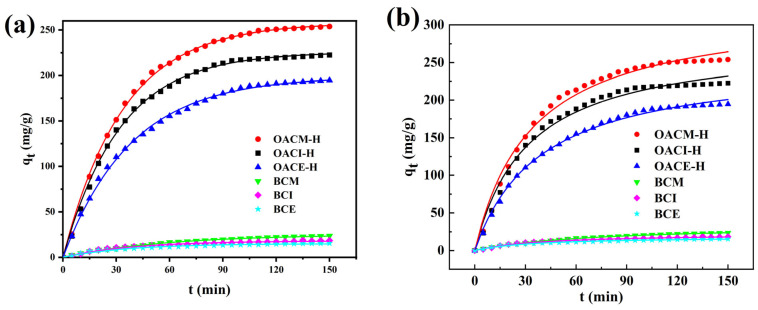
Sludge base kinetic curves for water vapor activation. (**a**) Quasi-primary-order kinetic curves, (**b**) quasi-second-order kinetic curves.

As illustrated in Figure 8, the adsorption kinetics of VOCs on the water vapor-activated sludge-derived biochar conform to both pseudo-first-order and pseudo-second-order models, with correlation coefficients (R^2^) greater than 0.97. The close agreement between fitted and experimental data suggests that both kinetic models effectively characterize the adsorption behavior. However, the adsorption capacity predicted by the pseudo-first-order model shows closer alignment with the experimental data, suggesting that this model offers a more precise representation of the adsorption process.

The adsorption rate constant *k*_1_ in the pseudo-first-order model (Table 9) follows the trend OAC-H > BC, aligning with the specific surface area trend observed in Figure 2a. This suggests that in the pseudo-first-order kinetic model, the adsorption rate is influenced by the biochar’s specific surface area, where a higher *k*_1_ value indicates stronger surface interactions, facilitating adsorption [36]. This is primarily attributed to the enhanced surface structure and the introduction of functional groups during the steam activation process, which improved the pore structure and increased the content of polar groups. These modifications facilitated the rapid entry of adsorptive molecules into the adsorption sites through physical interactions such as hydrogen bonding and van der Waals forces. In contrast, the original biochar had limited pore structures and fewer functional groups, resulting in slower adsorption processes that rely more on slow chemical interactions, leading to a lower adsorption rate [37].

#### 3.2.5. Isothermal Adsorption Modeling Analysis

**Figure 9:**
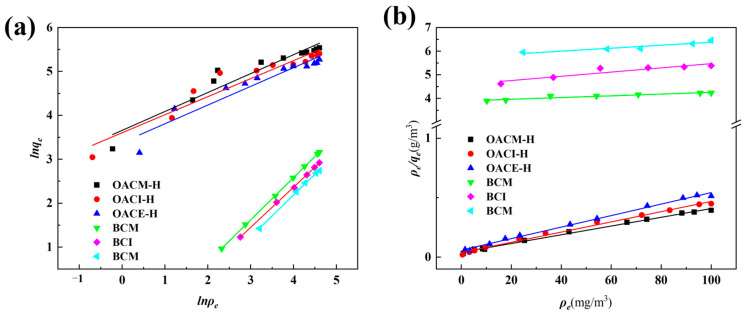
Fitted curve of isothermal adsorption of VOCs by biochar. (**a**) Freundlich isothermal adsorption curve, (**b**) Langmuir isothermal adsorption curve.

The adsorption data for water vapor-activated and unactivated SBC were fitted to the Langmuir and Freundlich models. Adsorption isotherms were analyzed using Origin 8.0 software, with the fitted results shown in Figure 9 and corresponding parameters listed in Table 10.

As shown in Figure 9, water vapor activation significantly enhanced the adsorption performance of SBC for methylbenzene, isopentane, and ethylene, and the OAC-H samples were superior to the unactivated BC samples throughout the adsorption process. Combined with the data in Table 6, the maximum adsorption capacities *q_m_* of the activated samples OACM-H, OACI-H, and OACE-H were 272.29, 233.01, and 206.82 mg/g, respectively, which were much higher than those of the unactivated samples BCM, BCI, and BCE, which were 24.12, 17.46, and 14.97, respectively, in Langmuir modeling mg/g, while the adsorption constant *k_L_* was also increased simultaneously, indicating that the activation enhanced the adsorption capacity of the monolayer [38]. In the Freundlich model, the 1/*n* values of the OAC-H series ranged from 0.41 to 0.43 (*n* ≈ 2.32–2.43), which were significantly lower than those of the BC series, which were close to 1.0 (*n* ≈ 1.05–1.08), indicating that the activated samples possessed stronger adsorption affinity and surface inhomogeneity. In addition, the *k_F_* values of the OAC-H series were as high as 29.47–38.80, which were much higher than those of the BC series (0.19–0.29). In summary, the water vapor activation not only enhanced the adsorption capacity, but also enhanced the adsorption ability on the inhomogeneous surfaces, so that the biochar displayed excellent adsorption performance at different concentrations of VOCs [39].

#### 3.2.6. Dualistic Competitive Adsorption Studies

At 25 °C with gas passing at 500 mL/min, 1.5 g of SBC was packed into the adsorption column to conduct competitive adsorption experiments for three binary VOC mixtures: methylbenzene–isopentane, methylbenzene–ethylene, and isopentane–ethylene.

**Figure 10:**
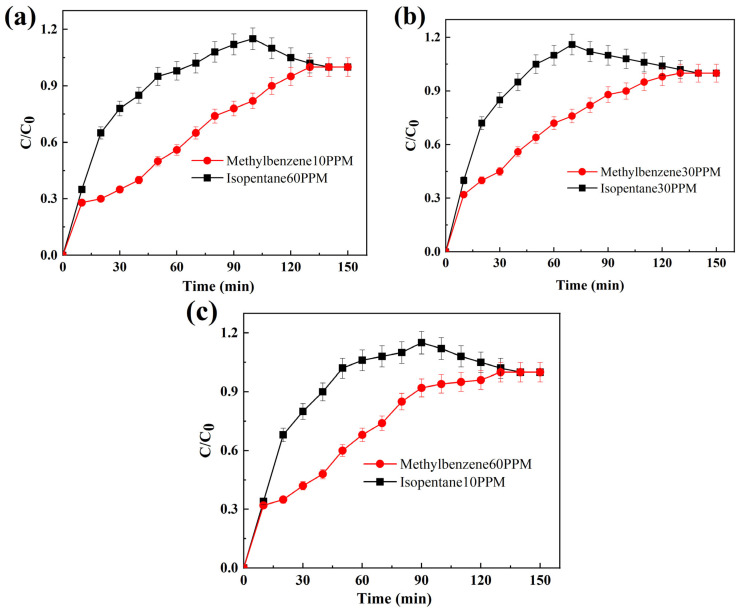
Adsorption penetration curves for methylbenzene and isopentane under varying initial concentrations. (**a**) Methylbenzene 10 PPM–isopentane 60 PPM, (**b**) methylbenzene 30 PPM–isopentane 30 PPM, (**c**) methylbenzene 60 PPM–isopentane 10 PPM.

The process of methylbenzene–isopentane binary competitive adsorption can be divided into three stages as shown in Figure 10a–c. The first stage is the initial stage, where methylbenzene and isopentane are uniformly adsorbed on the SBC. The second stage is the replacement stage; with the gradual increase of the adsorbed amount of methylbenzene with high boiling point in the adsorption column, the more volatile isopentane starts to desorb, which is manifested as the phenomenon of methylbenzene replacing isopentane, and the isopentane penetration curve appears to be peaked, i.e., the export concentration is briefly higher than that in the import. The third stage is the equilibrium stage, in which the methylbenzene adsorption is close to saturation, the replacement stops, and the adsorption of isopentane also tends to equilibrium. Under the condition of the same inlet concentration, isopentane with a low boiling point is easier to penetrate than methylbenzene with a high boiling point, and its penetration curve is above that of methylbenzene as a whole [40].

**Figure 11:**
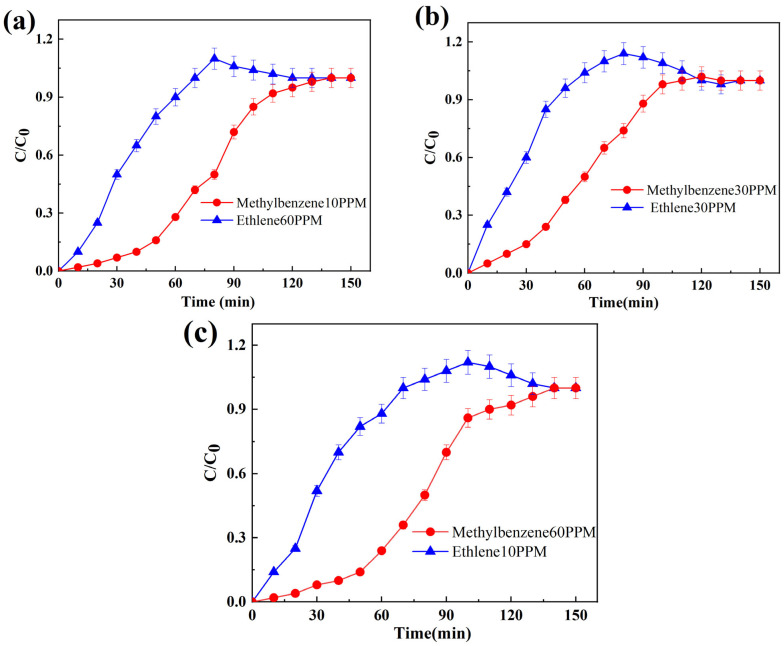
Adsorption penetration curves for methylbenzene and ethylene under varying initial concentrations. (**a**) Methylbenzene 10 PPM–ethylene 60 PPM, (**b**) methylbenzene 30 PPM–ethylene 30 PPM, (**c**) methylbenzene 60 PPM–ethylene 10 PPM.

Figure 11 shows that regardless of whether the concentrations of methylbenzene and ethylene were comparable, methylbenzene exhibited a consistently greater adsorption capacity compared to ethylene. In addition, ethylene exhibited a distinct peak in the breakthrough curve, indicating that at a certain point, its outlet concentration exceeded the inlet concentration. This suggests that ethylene, which had been previously adsorbed, underwent desorption. The desorption implies that methylbenzene, with its stronger adsorption affinity, displaced ethylene from the active sites on the SBC. Therefore, methylbenzene demonstrated superior adsorption performance compared to ethylene. In the binary competitive adsorption system of methylbenzene and ethylene, methylbenzene, as the stronger adsorbate, replaced ethylene during the adsorption process, leading to the peak observed in the ethylene breakthrough curve. Subsequently, both methylbenzene and ethylene were adsorbed until the biochar reached saturation. Furthermore, the breakthrough curves indicate that ethylene penetrated the biochar adsorption column earlier than methylbenzene, suggesting a weaker interaction between ethylene and the SBC.

**Figure 12:**
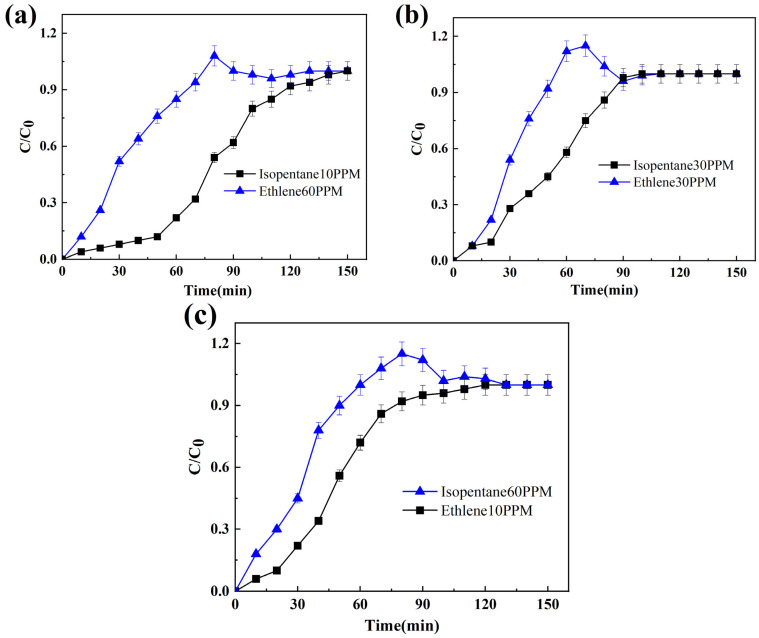
Adsorption penetration curves for isopentane and ethylene under varying initial concentrations. (**a**) Isopentane 10 PPM–ethylene 60 PPM, (**b**) isopentane 30 PPM–ethylene 30 PPM, (**c**) isopentane 60 PPM–ethylene 10 PPM.

As depicted in Figure 12, across three distinct sets of isopentane–ethylene concentrations, ethylene exhibited peaks on the penetration curves, indicative of the adsorption process. Displacement between ethylene and isopentane occurred, leading to adsorption at moments where the outlet concentration surpassed the inlet concentration. Isopentane demonstrated strong adsorption characteristics, whereas ethylene exhibited weaker adsorption tendencies. From the adsorption curves, it is evident that isopentane penetrated the adsorption column before ethylene, albeit with a lag. Subsequently, both isopentane and ethylene achieved adsorption saturation nearly simultaneously.

Adsorption behavior is influenced by molecular diameter, polarity, boiling point and molecular weight, with boiling point being the dominant factor. Experiments show that the adsorption capacity of methylbenzene > isopentane > ethylene is in the same order as its boiling point. In competitive adsorption, the greater the difference in boiling points, the more pronounced the substitution effect; e.g., methylbenzene can substitute isopentane and ethylene, and isopentane can substitute ethylene, with a peak in the permeation curve. Since adsorption is an exothermic reaction, the temperature rise reduces the adsorption capacity of low-boiling-point VOCs and strengthens the competitive advantage of high-boiling-point substances, which ultimately manifests itself in the substitution of strong adsorbates for weak adsorbates, resulting in a rise in the permeation curve of the latter.

The adsorption penetration curve analysis showed that the adsorption of VOCs on water vapor-activated SBC was closely related to its molecular weight [41]. Compounds with higher molecular weights were less sensitive to adsorption heat and had a weaker tendency to desorb, while compounds with lower molecular weights were susceptible to desorption by adsorption heat and returned to the gas stream, indicating that molecular weights significantly affected the adsorption stability and retention capacity.

The observed competitive adsorption sequence (methylbenzene > isopentane > ethylene) can be further elucidated by considering their molecular properties. Methylbenzene, as an aromatic compound, possesses a highly polarizable π-electron cloud and a relatively high molecular weight (92.14 g/mol) and boiling point (110.6 °C). These characteristics facilitate strong interactions with the adsorbent through multiple mechanisms: (1) π–π interactions with the graphitic structure of biochar, (2) potential dipole–dipole or hydrogen bonding interactions (O-H···π) with the oxygen-containing functional groups (e.g., -OH, -COOH) introduced by steam activation (as evidenced by FTIR), and (3) higher polarizability leading to stronger van der Waals forces. Isopentane, a branched alkane, has a lower boiling point (27.8 °C) and negligible permanent dipole moment, thus exhibiting weaker interactions primarily through dispersive forces (London forces) with the carbon surface. Its larger kinetic diameter compared to ethylene might contribute to slower diffusion but stronger van der Waals interactions than ethylene due to more atoms. Ethylene, a small unsaturated alkene, has the lowest boiling point (−103.7 °C), molecular weight (28.05 g/mol), and polarizability. Its double bond offers some potential for π-type interactions, but its small size and high volatility result in the weakest overall affinity and the most rapid breakthrough and displacement by higher-boiling-point VOCs (as seen in Figure 10, Figure 11 and Figure 12). While boiling point serves as a strong correlating macroscopic parameter indicative of volatility and intermolecular forces, the underlying adsorption strength is synergistically determined by a combination of factors: polarity/polarizability (influencing specific interactions like H-bonding and π–π stacking), molecular size/kinetics diameter (affecting micropore filling and diffusion rates), and molecular weight/polarity (influencing the strength of van der Waals forces). The steam-activated biochar, with its developed microporous structure and enriched oxygen-containing groups, provides a heterogeneous surface that exploits these differences, leading to the observed preferential and sequential adsorption.

## 4. Conclusions

(1) During the preparation of sludge–straw hybrid carbon, the biochar’s specific surface area and pore volume increased, while the average pore diameter decreased after carbonization and activation treatments compared to the raw materials. Nevertheless, the composition of functional groups on the biochar surface demonstrated remarkable stability, consistently featuring N-O, C-O-C, C=C, -OH, and various other functional groups.
(2) The ability of straw–sludge biochar to adsorb VOCs ranked as follows: methylbenzene showed the highest uptake, followed by isopentane, and finally ethylene. Water vapor activation significantly enhanced adsorption, with the optimized biochar group showing the highest efficiency, followed by the response surface group, single-feedstock biochar, and unactivated biochar. Notably, compared to the BC group, the maximum saturated adsorption capacities in the response surface group increased by 10.81 times for methylbenzene, 12.39 times for isopentane, and 12.36 times for ethylene. This represents increases of 112.21%, 74.53%, and 66.72% compared to the SAC group, and further improvements of 74.25%, 62.98%, and 55.25% over the CAC group. The adsorption properties of volatile organic compounds on sludge-derived biochar resembled chemisorption, closely mirroring the characteristics of monomolecular layer adsorption and highlighting the intricate dynamics involved.
(3) In competitive adsorption, the order of adsorption capacity magnitude of volatile organic compounds (VOCs) on water vapor-activated SBC was as follows: methylbenzene > isopentane > ethylene. Furthermore, the higher the boiling point, the higher the adsorption capacity. Additionally, the difference between boiling point values influences the adsorption strength, where compounds with higher boiling points displace those with lower boiling points more effectively.
(4) A blended biochar made from sludge and corn stover offers a cost-effective and sustainable alternative for removing VOCs. This adsorbent is suitable for treating exhaust gases rich in aromatic and aliphatic VOCs. Its applications include biomass power plants, chemical industrial parks, and wastewater treatment plants, offering a highly promising solution for achieving synergies between waste resource recovery and exhaust gas purification.

## Figures and Tables

**Table 1:** Proximate and ultimate analyses of sludge and corn stover.

	Sludge	Corn Stover
Proximate analysis (wt%)		
Moisture Content	93.4	4.8
Ash	38	8
Volatile Matter	17.38	74.98
Fixed Carbon	44.62	17.02
Ultimate analysis (wt%)		
C	30.7	41.49
H	4.71	5.7
N	20.94	43.67
O	5.01	1

**Table 2:** Levels of design variables.

Controlling Factor	Notation	Code Number		
		−1	0	1
Material proportion	A	0.25	0.50	0.75
Activation temperature (°C)	B	700	800	900
Activation time (min)	C	60	120	180

**Table 3:** Design and results of response surface tests.

Group	A	B (°C)	C (min)	Adsorption Capacity (mg/g)		
				Methylbenzene	Isopentane	Ethylene
MAC-1	0.50	800	120	254.41	221.13	194.08
MAC-2	0.50	800	120	253.24	223.74	195.71
MAC-3	0.50	800	120	254.98	220.46	196.24
MAC-4	0.50	800	120	254.93	219.26	196.45
MAC-5	0.50	800	120	255.46	220.23	194.59
MAC-6	0.50	700	60	201.25	190.76	181.67
MAC-7	0.50	900	60	231.78	211.67	189.68
MAC-8	0.50	900	180	237.56	214.79	194.59
MAC-9	0.50	700	180	220.68	208.68	175.04
MAC-10	0.75	800	60	224.35	194.87	174.74
MAC-11	0.75	900	120	243.4	207.89	190.35
MAC-12	0.75	800	180	241.65	209.14	185.91
MAC-13	0.75	700	120	197.77	187.18	174.8
MAC-14	0.25	800	60	178.98	170.71	168.63
MAC-15	0.25	800	180	189.87	177.58	158.91
MAC-16	0.25	900	120	172.86	177.47	175.5
MAC-17	0.25	700	120	169.11	160.55	165.45
SBC	-	-	-	23.62	18.57	15.48

**Table 4:** Tag explanation.

Notation	Explain
AC	Activated biochar
MAC	Response surface group mixed activated biochar
SAC	Sludge-based activated biochar
CAC	Corn stover activated biochar
OAC	Optimal activated biochar
SBC	Sludge-based biochar
CBC	Corn stover biochar
-H	H_2_O activation
M	Methylbenzene
I	Isopentane
E	Ethylene
MAC-1~17	Activated biochar from groups 1 to 17
MAC-H-1~17	Activated biochar prepared by H_2_O activation from groups 1 to 17
OACM	Optimal group activated biochar for methylbenzene adsorption
OACI	Optimal group activated biochar for isopentane adsorption
OACE	Optimal group activated biochar for ethylene adsorption

**Table 5:** Analysis of variance results for the RSM prediction of VOCs adsorption.

VOCs	Source	Sum of Squares	DF	Mean Square	F Value	*p*-Value Prob > F
Methylbenzene	Model	15,610.86	9	1734.54	2428.84	<0.0001
	A	4819.17	1	4819.17	6748.19	<0.0001
	B	1171.04	1	1171.04	1639.78	<0.0001
	C	356.44	1	356.44	499.12	<0.0001
	AB	438.48	1	438.48	614	<0.0001
	AC	10.27	1	10.27	14.38	0.0068
	BC	46.58	1	46.58	65.23	<0.0001
	A^2^	5597.8	1	5597.8	7838.5	<0.0001
	B^2^	2104.57	1	2104.57	2946.99	<0.0001
	C^2^	374.38	1	374.38	524.24	<0.0001
	Residual	5	7	0.7141		
	Cor. Total	15,615.86	16			
Isopentane	Model	6444.46	9	716.05	194.85	<0.0001
	A	1589.63	1	1589.63	432.57	<0.0001
	B	522.45	1	522.45	142.17	<0.0001
	C	222.39	1	222.39	60.52	0.0001
	AB	3.59	1	3.59	0.9772	0.3558
	AC	13.69	1	13.69	3.73	0.0949
	BC	54.76	1	54.76	14.9	0.0062
	A^2^	3311.85	1	3311.85	901.22	<0.0001
	B^2^	391.75	1	391.75	106.6	<0.0001
	C^2^	98.77	1	98.77	26.88	0.0013
	Residual	25.72	7	3.67		
	Cor. Total	6470.19	16			
Ethene	Model	2351.89	9	261.32	114.6	<0.0001
	A	410.55	1	410.55	180.05	<0.0001
	B	353.25	1	353.25	154.92	<0.0001
	C	0.0091	1	0.0091	0.004	0.9514
	AB	7.56	1	7.56	3.32	0.1114
	AC	109.1	1	109.1	47.85	0.0002
	BC	33.29	1	33.29	14.6	0.0065
	A^2^	1083.73	1	1083.73	475.27	<0.0001
	B^2^	34.1	1	34.1	14.95	0.0062
	C^2^	225.81	1	225.81	99.03	<0.0001
	Residual	15.96	7	2.28		
	Cor. Total	2367.85	16			

**Table 6:** Residual evaluation and statistical validation of the regression model.

Statistical Projects	Value		
	Methylbenzene	Isopentane	Ethene
Std.Dev	0.85	1.92	1.51
Mean	222.49	200.95	183.08
C.V%	0.38%	0.95%	0.82%
PRESS	38.43	246.56	193.28
R-Squared	0.9997	0.9960	0.9933
Adj R-Squared	0.9994	0.9909	0.9846
Pred R-Square	0.9975	0.9619	0.9184
Adeq Precisior	131.132	40.075	30.900

**Table 7:** Ideal process parameters.

Types of VOC	Ratio	Activation Temperature (°C)	Activation Time (min)	Predicted Amount of Adsorption (mg/g)	Actual Amount of Adsorption (mg/g)	Relative Error (%)
methylbenzene	0.60	868.48	123.95	259.57	253.84	2.26
isopentane	0.60	855.71	157.85	224.43	222.57	0.84
ethene	0.62	847.65	139.56	198.44	194.63	1.96

**Table 8:** Surface area, pore capacity, and mean pore diameter of biochar derived from sewage sludge.

Sorbent	Specific Surface Area (m^2^/g)	Micropore Volume (cm^3^/g)	Mesopore Volume (cm^3^/g)	Total Pore Volume (cm^3^/g)	Average Pore Diameter (nm)
OACM-H	158.97	0.0748	00.0691	0.1509	2.5864
OACI-H	133.64	0.0631	0.0684	0.1396	3.3843
OACE-H	134.73	0.0285	0.1151	0.1436	3.2082
SAC	82.53	0.0343	0.0254	0.0846	5.0123
CAC	80.71	0.0131	0.0265	0.0803	4.9854
SBC	45.29	0.0038	0.0133	0.0456	7.0577

**Table 9:** Kinetic parameters of VOCs adsorption on SBC.

Adsorbent	Quasi-First-Order Kinetic Model			Quasi-Second-Order Kinetic Model		
	*q_e_* (mg/g)	*k*_1_	R^2^	*q_e_* (mg/g)	*k*_2_	R^2^
OACM-H	257.9886	0.0295	0.9977	323.6855	0.00009	0.9880
OACI-H	225.8140	0.0309	0.9978	280.2891	0.00013	0.9831
OACE-H	198.8720	0.0260	0.9988	252.7448	0.00011	0.9968
BCI	18.0388	0.0255	0.9833	22.9060	0.00111	0.9882
BCE	15.0130	0.0273	0.9798	18.6979	0.00149	0.9921
BCM	25.4021	0.0170	0.9979	35.0140	0.00040	0.9987

**Table 10:** Fitting parameters for the isothermal adsorption by straw–sludge biochar.

Adsorbent	Freundlich Isothermal Adsorption Model			Langmuir Isothermal Adsorption Model		
	*k_F_*	*n*	R^2^	*q_m_*	*k_L_*	R^2^
OACM-H	38.7992	2.3217	0.9219	272.2910	0.0888	0.9950
OACI-H	36.7375	2.4348	0.9215	233.0053	0.1098	0.9929
OACE-H	29.4679	2.3495	0.9128	206.8158	0.0815	0.9943
BCM	0.2924	1.0455	0.9994	24.1215	0.0137	0.9732
BCI	0.2641	1.0784	0.9996	17.4635	0.0244	0.9638
BCE	0.1879	1.0613	0.9992	14.9654	0.0258	0.9583

## Data Availability

The original contributions presented in this study are included in the article. Further inquiries can be directed to the corresponding author.
